# Use of low-threshold naloxone boxes for opioid overdose prevention in a Midwestern US State: a public health program evaluation

**DOI:** 10.1186/s12954-025-01333-6

**Published:** 2025-11-18

**Authors:** Pamela S. Lynch, Lou Gamalski, Virginia Roys, Autumn Albers, Katie Burk, Sara Durán, Shelley N. Facente

**Affiliations:** 1 Harm Reduction Michigan, 124 Munson Avenue, Traverse City, MI 49686 USA; 2 Facente Consulting, 6500 Fairmount Ave, Suite 10, El Cerrito, CA 94530 USA; 3 Division of Community Health Sciences, School of Public Health, University of California, Berkeley, 2121 Berkeley Way West # 5302, Berkeley, CA 94720 USA

**Keywords:** Naloxone, Opioid, Overdose, Substance use intervention, harm reduction

## Abstract

**Introduction:**

Despite reductions in overdose deaths reported nationally in 2025, overdose remains a leading cause of death in Michigan and the broader United States. Naloxone is a safe and highly effective opioid antagonist that can reverse opioid overdose, and community-based distribution to people at highest risk of overdose is a key overdose death prevention strategy.

**Methods:**

In 2021, Harm Reduction Michigan (HRMI) launched an innovative naloxone box model to boost community-based naloxone distribution through publicly accessible, unlocked, outdoor naloxone boxes. To evaluate HRMI’s naloxone box model, we conducted stakeholder interviews and analyzed secondary quantitative data about naloxone box stocking and placement.

**Results:**

As of December 2024, HRMI has placed 184 naloxone boxes in 85 jurisdictions within 47 Michigan counties, resulting in 24,428 doses of naloxone distributed from 2023 to 2024 alone. Naloxone boxes are prevalent in some, but not all, counties with high overdose death rates, suggesting the need for data-driven placement to support equitable access. However, stakeholders universally perceived the naloxone box model as impactful and crucial to saving lives, noting that naloxone boxes democratize naloxone distribution through their low-barrier, 24/7 availability and relative anonymity. They noted that amid persistent drug-related stigma, naloxone boxes create opportunities for productive conversations about overdose, drug use, and harm reduction in communities.

**Conclusion:**

These models are strengthened by partnership with non-traditional partners (such as restaurants or retail stores) who request to host boxes, along with meaningful involvement of people with lived experience of drug use, overdose, and interrelated conditions in box planning, implementation, and maintenance.

**Supplementary Information:**

The online version contains supplementary material available at 10.1186/s12954-025-01333-6.

## Introduction

Despite reductions in overdose deaths reported nationally in 2025, overdose remains a leading cause of death in Michigan and the broader U.S [1, 2]. In 2023, Michigan was home to 2,931 opioid-related overdose deaths, with an age-adjusted death rate of 29.2 deaths per 100,000 residents [3]. During the fatal overdose epidemic, Michigan has also observed notable racial/ethnic disparities. In 2023, the overdose-related death rate among American Indian or Alaska Native, non-Hispanic residents was twice that of white non-Hispanic residents, and the death rate among Black non-Hispanic residents was nearly three times that of white non-Hispanic residents [3].

A range of evidence-based strategies are recommended to prevent overdose. Several strategies include the distribution of naloxone, which is a safe and highly effective opioid antagonist that can reverse opioid overdose [4]; however, many of the strategies used and evaluated to date have been time-intensive opioid education and naloxone distribution (OEND) initiatives from within syringe services programs [5–9]; programs to distribute naloxone to first responders [10, 11], librarians [12], or teachers [13]; or expensive naloxone vending machines [14–17], which provide ready access but at great cost to establish and maintain. The most effective strategies have been shown to be those designed to distribute naloxone directly to people at highest risk of overdosing, with little to no restriction [8, 18]. Low-threshold, community-based naloxone distribution is especially important to (i) ensure naloxone ends up in the hands of the people most likely to be near someone who is overdosing, and (ii) achieve naloxone saturation [19]—a state where there is sufficient naloxone availability to maximize a person’s chance of survival during an overdose.

**Fig. 1:**
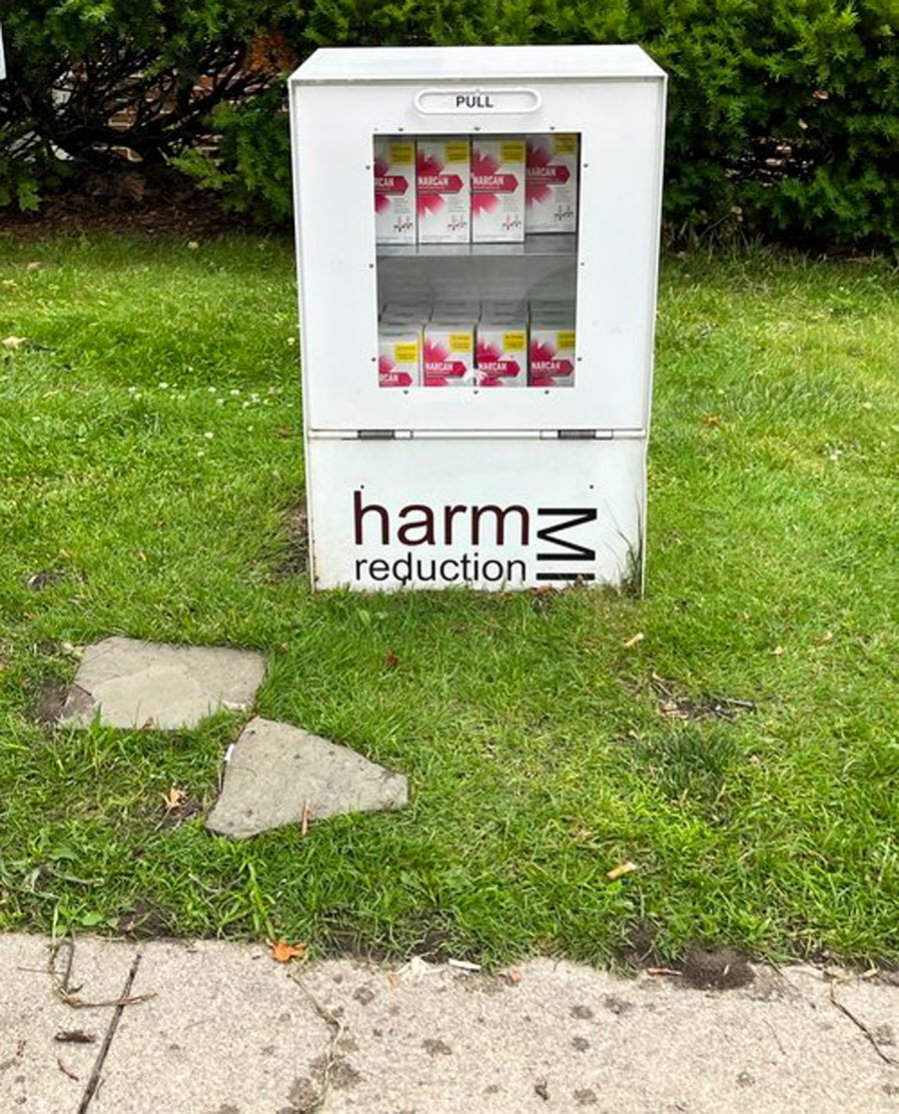
Photo of a Harm Reduction Michigan community naloxone box

In 2021, Harm Reduction Michigan (HRMI) launched an innovative naloxone box model to boost community-based naloxone distribution and strive toward naloxone saturation. In this model, HRMI placed publicly accessible, unlocked boxes (Fig. 1) where naloxone can be stocked upon request near clinics, pharmacies, businesses, government agencies, and other partner sites. The naloxone box model reinvents the concept of harm reduction vending machines—which have similar intent and have been found to be effective [17, 20, 21]—with a less expensive, easily transportable alternative that is not dependent on electricity or a wifi connection.

HRMI is a health equity and recovery-focused community-based organization in the state of Michigan. Founded in 2016, HRMI currently hosts drop-in locations and outreach services for people who use drugs in seven municipalities. In addition to its various efforts to support naloxone distribution, HRMI’s overdose prevention services also include syringe access and disposal, training in overdose recognition and response, medications for opiate use disorder (MOUD) and linkage of clients to health and social services. In the naloxone box model, the naloxone is obtained at no cost through the Michigan Department of Health & Human Services, paid for with state opioid response grants and opioid settlement funds. As the model is designed to allow for 24/7 access for a person needing naloxone, boxes are placed outdoors, in unrestricted areas. While there was an effort to place boxes in parts of the state without functioning syringe services programs (e.g., more remote areas) and centrally located in communities with large numbers of people who use drugs (e.g. libraries, liquor stores), in general boxes have been placed in response to community partner request, wherever their location. After request of a community partner, HRMI places each box in a public place they specify, and then an onsite point person backed up by HRMI staff monitors stock and restocks the naloxone boxes with the state-provided naloxone as needed. The program launched in 2021, and as of December 2024 HRMI was supporting 184 boxes—each holding between 3 and 6 dozen doses of naloxone when fully stocked—in 85 jurisdictions in 47 counties.

To better understand the reach and impact of this naloxone box model, HRMI contracted with Facente Consulting, an equity-focused public health consulting firm with expertise in both impact evaluation strategies and harm reduction interventions. This program evaluation was intended to assess whether HRMI’s naloxone box program had demonstrated proof of concept as a feasible, sustainable, and seemingly effective model of naloxone distribution, and generate some lessons learned or insights to support program scale-up or replication.

## Methods

### Analysis of quantitative secondary data

First, HRMI exported naloxone box data from the SSP Utilization Platform (SUP) hosted by the State of Michigan’s Department of Health & Human Services [22]. This platform, which reflects data collected and entered by HRMI staff, includes quarterly data on naloxone doses distributed for each naloxone box location. Facente Consulting then cleaned the SUP data and analyzed it using descriptive statistics. In addition, we inductively categorized each location site where naloxone boxes are stationed into one of the following categories: (1) Cannabis Dispensary, (2) Church, (3) Department of Public Health, (4) Harm Reduction, (5) Healthcare, (6) Public Services, (7) Retail, (8) Social Services, (9) Substance Use Disorder Recovery, (10) Restaurant, Coffee Shop, or Bar, and 11) Other. Finally, we used the ArcGIS platform to visualize the distribution of naloxone boxes spatially and to explore potential relationships between naloxone boxes, county population sizes [23], and county overdose death data from the Michigan Overdose Data to Action data dashboard (provisional 3-year average overdose death rate and count data, 2021–2023) [3].

### Qualitative data collection and analysis

To achieve a deeper understanding of how the naloxone box program was experienced by people involved in program implementation, Facente Consulting developed a structured interview guide with input from HRMI. Questions explored (1) the interviewee’s role in overdose prevention efforts in Michigan overall and in the naloxone box program specifically, (2) their reflections on the successes and weaknesses of the naloxone box program, (3) their lessons learned and ideas for future priorities of the program, and (4) any key stories of naloxone from the boxes being used to reverse overdoses. (The interview guide is available in Supplemental Material.) Interviewees were chosen by HRMI to represent a variety of partners who were involved in the naloxone box program model, representing different naloxone box program locations as well as staff who helped launch the program. Using that list we conducted 10 in-depth interviews with 11 people involved in naloxone box programming, including three current and former staff members working on program implementation, four people involved in hosting and restocking boxes at the non-traditional sites where they work (a library, cannabis dispensary, community space, and party store), three health department staff involved in program administration, and a person who had personally used the boxes to access naloxone. More than half of those interviewed voluntarily disclosed their own history of substance use. Interviews were conducted via Zoom [Zoom Video Communications, Inc., San Jose, CA] and lasted approximately 30–60 min. Interviews were recorded via the Zoom recording feature with participants’ consent, and audio recordings were transcribed using the otter.ai platform [Otter.ai, Inc., Mountain View, CA], followed by manual review and transcript editing by the interviewer to ensure accuracy. Two Facente Consulting staff members then developed and iterated a codebook, applying a deductive coding approach focused on thematic analysis. Discrepancies in coding were resolved by consensus.

### Ethics

As this was a quality improvement-focused evaluation for public health practice and not human subjects research [24], no IRB approval was obtained.

## Results

Since the first pilot naloxone box was placed in Baldwin County, Michigan in 2021, HRMI has continued to expand the program annually. As of December 31, 2024, HRMI had placed 184 naloxone boxes in 85 jurisdictions within 47 Michigan counties (57% of all counties), resulting in 24,428 doses of naloxone distributed from 2023 to 2024 alone. Of the 184 boxes, HRMI is responsible for stocking 130, while partners stock the remaining 54.

**Fig. 2:**
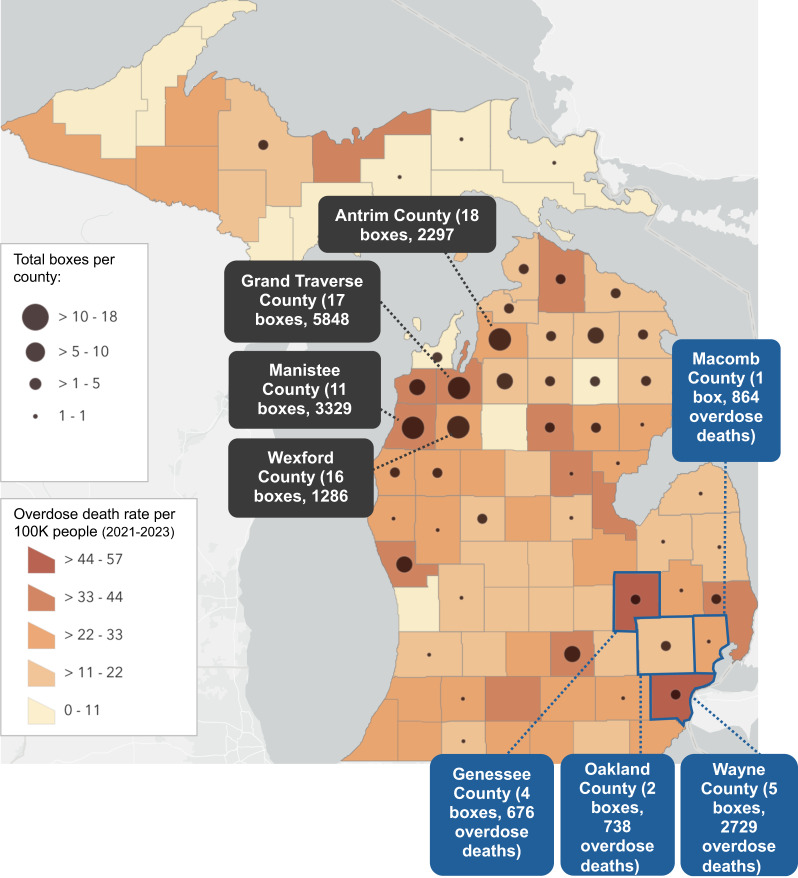
The 47 Michigan counties with at least one naloxone box are represented by black dots. Larger dots correspond to more naloxone boxes. Antrim, Grand Traverse, Wexford, and Manistee counties (see dark grey labels) host the most boxes and doses. The color of each county represents overdose death *rate*, with darker shading reflecting higher overdose death rates. The blue outlined counties are the counties with the highest overdose death *counts*; these counties also are home to approximately 80% of Michigan’s Black population

As shown in Fig. 2, some counties with high overdose death rates (darker orange/red colors) have one or more naloxone boxes (black dots), while others have no boxes. Data tables detailing the number of boxes and doses per county along with overdose data (death rate per 100,000 people and absolute counts of overdose deaths) per county can be found in Supplemental Table 1. Data suggesting that box placement may not be reaching those who need it the most was reinforced by interviewee perspectives. Interviewed stakeholders acknowledged that a data-driven approach to naloxone box placement could further equitable distribution efforts, including racial equity and equity in naloxone access among people living in lower-income, rural counties.

**Fig. 3:**
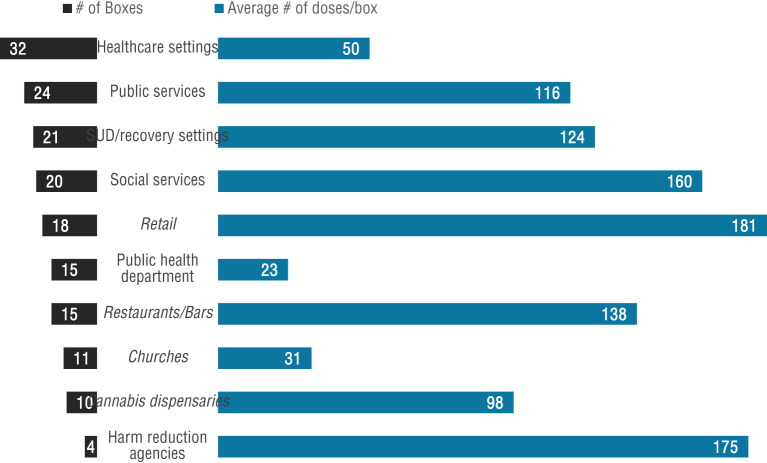
The number of boxes in each site type (left side), along with the average doses per box for that site type (right side). Nontraditional partners (retail, restaurants, churches, and cannabis dispensaries) are italicized. 14 boxes at sites categorized as “other” type (averaging 45 doses/box) are not shown

Naloxone boxes were most commonly implemented in healthcare settings and public service sites, with 32 and 24 boxes at those sites, respectively (Fig. 3, left side). While SUD recovery facilities had the 3rd highest number of boxes, more than 2/3 of doses were placed at just one site in Midland that included SUD-related and other services. There were also many nontraditional partner sites with boxes, such as retail locations, churches, cannabis dispensaries, and restaurants, coffee shops, and bars.

When considering total naloxone doses distributed through the boxes, restaurants and retail sites—both non-traditional partners—topped the list, with 3,740 and 3,261 doses, respectively. When considering average doses distributed per box (Fig. 3, right side), retail settings and harm reduction settings ranked highest, with 181 and 175 doses per box, respectively. Notably, the site with the most doses distributed (city of Midland) was based near multiple human services providers, including health department services and a SUD family support organization.

Stakeholders who were asked about box placement during interviews shared the importance of placing boxes in visible, welcoming, low-threshold places, noting that people are less likely to take naloxone if they perceive risk or judgment. As one person explained,

*Placement really matters; it’s very important where we place these. Putting it at a place like Catholic Human Services*,* you might as well not even place it…Or if it’s going to be like right in front of a police station. That’s probably not a good idea. I even have one in front of a fire station*,* and some people are a little leery about that until it gets a little darker.*

One of the most common successes of the naloxone box model described by interviewees was that it saves lives. Interviewees underscored the model’s life-saving potential, such as this worker at a non-traditional site hosting a box, who explained:

*It’s pretty intense when somebody mentions that they’re familiar with somebody’s life who was saved because [the naloxone box] was there. The fine line between life and death in some circumstances…it’s extremely profound and completely confirms every reason in the world to have these things out there.*

Some also shared personal stories of people whose lives were saved because of a naloxone box, like this interviewee:

*There was a grandma that would come…and she said that her granddaughter was using and everybody knew it. She was worried. [We] had the conversation about leaving the bathroom door unlocked…[get] permission to check on them if they’ve been in the bathroom. She comes back two weeks later saying that she reversed her granddaughter on the bathroom floor.*

Interviewed stakeholders saw the naloxone boxes as generally cost-effective and sustainable. Because the State of Michigan provides free naloxone to HRMI for this program, the main cost is the personnel time for implementation support, provided by community partners. One interviewee recalled:

*We immediately saw*,* you know*,* the benefits of these boxes. They don’t require electricity. They’re much smaller than a vending machine. They’re much cheaper than a vending machine. I can pick one up and put it in my trunk. I can pick one up and put it in my passenger seat. If we have a placement that needs to go in and I don’t have anybody else to deliver it*,* I can do it.*

Notably, the boxes only work to the extent that they are monitored and restocked, which HRMI staff acknowledged as one possible sustainability challenge: limited HRMI staff and community partner bandwidth may constrain how much the model can sustainably grow. They did report that the program was slow to start, and that maintaining boxes required them to observe, listen, and respond to concerns and adapt methods or systems to meet the needs of community partners. However, interviewees generally reported that anticipated logistical challenges—such as box vandalism, theft of the boxes for sheet metal value, or issues related to naloxone freezing in the cold Michigan winter—have not occurred, reducing the maintenance burden on staff below what was expected.

Interviewees acknowledged that while logistical issues with the boxes have not posed the challenge they anticipated, stigma and misinformation are persistent barriers to naloxone access, even within programs that support people who use drugs. They shared that the naloxone box model opens up opportunities for education and communication about overdose prevention and response, as one person working at a non-traditional site hosting a box said:

*We’ve had a lot of opportunities to have conversations with people who start out the conversation by not thinking that it’s really that great of an idea to have it available*,* and then by the end of the conversation*,* they realize that it’s actually a great idea…It’s totally changed their mind…. I think we’ve had a lot of success with people in terms of broadening their horizons on what the purpose really is.*

Some interviewees did cite initial community pushback, for example:

*I was there*,* with like*,* the first 100 or so that were put out. You know*,* one person in one town put a bunch of post-it notes with all sorts of ‘this is enabling drug use’ type messaging on there.*

In another case, a local librarian had picked up a box for her community, and recalled:

*I picked up the box in Traverse City*,* and all the Narcan*,* and I delivered it to the Chamber of Commerce*,* and then*,* like*,* five days later*,* it just showed up at the library with no explanation. And it turned out that they didn’t want it*,* because that “wasn’t the look they were going for in town.”*

Despite these types of stories, interviewees consistently noted that the boxes had successfully led to changes in community sentiment, with more and more positive feedback over time. Several stakeholders perceived that more community members were seeing the need for increased access to low-barrier naloxone, and thus requested to host their own box. For example:

*In the first year*,* it seemed like people didn’t know what* [naloxone] *was*,* and it had a bad name—people didn’t like seeing it in their city. At first*,* they’re like*,* ‘Oh*,* this makes our city look like we have a drug problem.’ That’s something that I’ve heard over and over again— not as much these days*,* but in the beginning.*

However, interviewees also emphasized the importance of being prepared and positioned to have conversations and share materials (e.g., brochures) to create understanding and buy-in for naloxone boxes. Sharing personal stories of how overdose has impacted oneself or one’s community were noted as powerful ways to bring others into the conversation, build empathy, and increase the likelihood of support.

## Discussion

In the first 3 years of its naloxone box model, HRMI has demonstrated proof of concept—that community members will partner in placing and restocking the boxes, that they are being utilized, and that they have value in the community, likely leading to lives being saved through overdose reversal. HRMI developed this naloxone box model as a community-driven response to the challenges of the vending machine model that has been shown to be effective [17, 20, 21], but requires a power source, is expensive, and is too heavy to easily move. The ease with which boxes can be set up and maintained as a major advantage of the model.

Among diverse naloxone box settings, both traditional trusted partners and non-traditional partners have been key in distributing naloxone doses. However, in this pilot, geographic distribution was not data driven, and was initially solely by request from a community partner, which posed a challenge to achieving the goal of naloxone saturation in the areas with greatest need. The initial expansion of boxes, which occurred organically outwards from HRMI’s home base in Grand Traverse County upon request by community partners, had unintended racial equity implications. Grand Traverse County is located in the Northern Lower Peninsula of Michigan, a region with a relatively high proportion of white residents [25]. Overdose death rates among Black Michigan residents are nearly triple that of white residents [3], and the four counties with the highest overdose death counts house approximately 80% of Michigan’s Black population [25]. However, these four counties (outlined in blue in Fig. 2) have relatively few naloxone boxes. A data-driven approach to box placement would increase naloxone access in areas of highest need, including counties with large proportions of Black residents, and low-income rural counties. Moving in this data-driven direction will require ongoing partnership between HRMI and the Michigan Department of Health & Human Services, as well as efforts to meaningfully engage and support buy-in within communities that would benefit from new naloxone boxes.

To achieve naloxone saturation (in which a person experiencing an overdose has a high chance that naloxone will be used to reverse the overdose and prevent death), the most critical step is to get naloxone out into the community in need [19]. The naloxone box model has democratized naloxone distribution and furthered naloxone saturation in an anonymous and low-threshold way, aligned with harm reduction principles [26]. Through the outdoor boxes, naloxone is available 24 hours a day in a wide range of community settings. While several interviewees noted that a cross-section of community members accessed naloxone through the boxes, there are some specific populations that may be more likely to access naloxone through a low-threshold box and not from any other sources, including (i) summer visitors who may not have access to naloxone at home, (ii) people who seek anonymity, including those on probation, parole, and in recovery, (iii) people experiencing homelessness, (iv) people who sell drugs, and (v) people who otherwise might not feel comfortable accessing naloxone from brick-and-mortar agencies due to stigma and shame.

Notably, overdose death rates in Michigan declined nearly 5 times faster than the U.S. average between 2021 and 2023 [3, 27]—the same years when the HRMI naloxone box model was scaling. Given the low threshold nature of naloxone boxes—in which overdose reversal is not formally tracked—causal links cannot be established between HRMI’s programming and overdose death trends. However, statewide trends can help contextualize the impact of the naloxone box model. Indeed, third-party analyses of the state’s overdose death decline have emphasized community-based naloxone distribution and naloxone saturation as key contributors [28].

There are a few main limitations to our analysis. First, HRMI staff bandwidth is typically filled supporting the direct needs of the community; this means that SUP data entry is not always able to be prioritized and reported naloxone dose data are likely underestimates. Second, more doses of naloxone at a given box may not necessarily indicate a greater community uptake of the intervention, as such boxes may simply be stocked more consistently and/or have more staff or community partner support for restocking. Third, this evaluation was not designed to capture data on actual overdose reversals as a result of naloxone obtained through this program, and thus we were not directly able to assess effectiveness of the intervention. Finally, during the qualitative interviews we did not directly interview people who are unsupportive of the program, though some interviewees described examples of community pushback that they had personally witnessed.

In summary, there are six key lessons learned from HRMI’s experience implementing naloxone boxes:

1. Local champions are paramount: Launching and sustaining a naloxone box requires local champions responsible for the box who can generate buy-in from their local community. People with lived experience make especially strong champions.
2. Non-traditional partners confer substantial program value: Retail and restaurant sites distributed the most doses of naloxone, demonstrating the importance of engaging non-traditional partners not typically involved in harm reduction in naloxone distribution.
3. Strategic box placement is key to success: Identifying local settings that are visible, welcoming, and trusted by people who use drugs makes it more likely that boxes will be utilized.
4. Community partners play a crucial role in destigmatizing harm reduction: Given persistent stigma related to drug use, community partners who set up or restock naloxone boxes must lead conversations about overdose prevention to build empathy and support for evidence-based naloxone distribution.
5. Naloxone box roll-out requires persistence and nimbleness: Stakeholders acknowledged that getting boxes started was challenging and that maintaining them required adjustment, resilience, and patience along the way.
6. Organic placement may not support equitable access: While the HRMI model has been successful in scaling, the organic expansion of the program did not maximize box implementation in areas of Michigan with the most overdose, including predominantly Black counties and lower-income rural counties further from Grand Traverse County.

## Supplementary Information

Supplementary Material 1.

Supplementary Material 2.

## Data Availability

Data downloaded from the State of Michigan’s SSP Utilization Program is publicly accessible, but can also be obtained from the corresponding author on reasonable request.

## Abbreviations

HRMI: Harm Reduction Michigan
MOUD: Medications for Opioid Use Disorder
SSP: Syringe Services Program
SUP: SSP Utilization Program
SUD: Substance Use Disorder
