# Supplementary material for: Use of low-threshold naloxone boxes for opioid overdose prevention in a Midwestern US State: a public health program evaluation
Source: Harm Reduct J. 2025 Nov 18;22:185. doi: 10.1186/s12954-025-01333-6 (PMC12625459; doi:10.1186/s12954-025-01333-6)
Supplement: Supplementary file 1 — Supplementary Material 1. [file 12954_2025_1333_MOESM1_ESM.docx]

HRMI Naloxone Box Interview Guide

*Interviewee role and experience*

1. How are you involved in overdose prevention efforts in Michigan?
   1. (if applicable) Where do you work?
2. In what ways are you involved with the naloxone boxes?
   1. How did you become involved locally?
   2. What can you tell me about the history of the program overall?

*Reflections on successes and areas of growth*

1. How do community members learn about naloxone boxes in the area?
2. What populations/groups do you think are accessing naloxone via the naloxone boxes that aren’t getting naloxone in other ways (e.g. via SSPs, pharmacies, doctors, etc.)?
3. In what ways have the naloxone boxes been a success?
4. What concerns do you have about the effectiveness of the naloxone box model?
5. What is your perspective in terms of how well the boxes are stocked and restocked?
   1. What is your take on the sustainability of these efforts?

*Ideas of lessons learned and future priorities*

1. What are your thoughts on how to grow the naloxone box program?
   1. What populations should be targeted? What types of settings? What locations?
2. What are your lessons learned about the naloxone box program thus far?

*Stories*

1. Have you heard stories about naloxone from the boxes being used to reverse overdoses? If so, which ones have stuck with you?
