# Supplementary material for: Use of low-threshold naloxone boxes for opioid overdose prevention in a Midwestern US State: a public health program evaluation
Source: Harm Reduct J. 2025 Nov 18;22:185. doi: 10.1186/s12954-025-01333-6 (PMC12625459; doi:10.1186/s12954-025-01333-6)
Supplement: Supplementary file 2 — Supplementary Material 2. [file 12954_2025_1333_MOESM2_ESM.docx]

# **Supplemental Table 1:** **County Boxes and Overdose Death Data (for counties with boxes)**

The next two pages show the number of naloxone boxes per county, the total naloxone doses distributed in those boxes 2023–2024, and the overdose death rate/count per county (average provisional overdose data 2021–2023), in alphabetical order by county.

| **County Name** | **# of boxes** | **# of doses**  (2023–2024) | **Overdose death rate per 100K** (2021–2023) | **# of overdoses** |
| --- | --- | --- | --- | --- |
| Alcona County, Michigan | 5 | 48 | 19.2 | 6 |
| Allegan County, Michigan | 1 | 48 | 13.48 | 49 |
| Alpena County, Michigan | 5 | 478 | 15.02 | 13 |
| Antrim County, Michigan | 18 | 2297 | 23.37 | 17 |
| Arenac County, Michigan | 1 | 0 | 24.3 | 11 |
| Benzie County, Michigan | 9 | 1184 | 34.61 | 19 |
| Charlevoix County, Michigan | 4 | 223 | 21.55 | 17 |
| Cheboygan County, Michigan | 3 | 600 | 35.98 | 28 |
| Chippewa County, Michigan | 1 | 48 | 7.35 | 8 |
| Crawford County, Michigan | 5 | 444 | 19.77 | 8 |
| Emmet County, Michigan | 3 | 96 | 11.71 | 12 |
| Genesee County, Michigan | 4 | 0 | 56.06 | 676 |
| Gladwin County, Michigan | 1 | 0 | 34.98 | 27 |
| Grand Traverse County, Michigan | 17 | 5848 | 20.04 | 58 |
| Huron County, Michigan | 1 | 0 | 19.2 | 18 |
| Ingham County, Michigan | 9 | 864 | 40.24 | 343 |
| Iosco County, Michigan | 1 | 48 | 32.65 | 25 |
| Kalamazoo County, Michigan | 1 | 96 | 23.61 | 185 |
| Kalkaska County, Michigan | 8 | 1754 | 20.17 | 11 |
| Kent County, Michigan | 1 | 48 | 17.95 | 355 |
| Lake County, Michigan | 2 | 582 | 26.47 | 10 |
| Lapeer County, Michigan | 1 | 96 | 24.4 | 65 |
| Leelanau County, Michigan | 2 | 180 | 10.2 | 7 |
| Luce County, Michigan | 1 | 0 | 0 | 1 |
| Macomb County, Michigan | 1 | 48 | 32.94 | 864 |
| Manistee County, Michigan | 11 | 3329 | 38.23 | 29 |
| Marquette County, Michigan | 2 | 48 | 13.5 | 27 |
| Mason County, Michigan | 3 | 192 | 27.2 | 24 |
| Mecosta County, Michigan | 2 | 120 | 13.1 | 16 |
| Midland County, Michigan | 1 | 1840 | 12.75 | 32 |
| Montmorency County, Michigan | 9 | 384 | 20.9 | 6 |
| Muskegon County, Michigan | 6 | 156 | 39.46 | 209 |
| Newaygo County, Michigan | 1 | 48 | 23.58 | 36 |
| Oakland County, Michigan | 2 | 0 | 19.38 | 738 |
| Oceana County, Michigan | 1 | 48 | 27.19 | 22 |
| Ogemaw County, Michigan | 2 | 0 | 25.43 | 16 |
| Oscoda County, Michigan | 2 | 48 | 0 | 3 |
| Otsego County, Michigan | 4 | 264 | 16.9 | 13 |
| Presque Isle County, Michigan | 3 | 192 | 14.97 | 6 |
| Roscommon County, Michigan | 3 | 96 | 37.96 | 27 |
| Sanilac County, Michigan | 1 | 48 | 16.4 | 20 |
| Schoolcraft County, Michigan | 1 | 0 | 0 | 4 |
| St. Clair County, Michigan | 2 | 48 | 33.09 | 159 |
| Tuscola County, Michigan | 1 | 48 | 15.11 | 24 |
| Washtenaw County, Michigan | 1 | 120 | 23.29 | 256 |
| Wayne County, Michigan | 5 | 1083 | 51.77 | 2729 |
| Wexford County, Michigan | 16 | 1286 | 27.29 | 28 |
